# Assessing the Applicability of E-Therapies for Depression, Anxiety, and Other Mood Disorders Among Lesbians and Gay Men: Analysis of 24 Web- and Mobile Phone-Based Self-Help Interventions

**DOI:** 10.2196/jmir.3529

**Published:** 2014-07-03

**Authors:** Tomas Rozbroj, Anthony Lyons, Marian Pitts, Anne Mitchell, Helen Christensen

**Affiliations:** ^1^Australian Research Centre in Sex, Health and SocietyFaculty of Health SciencesLa Trobe UniversityMelbourneAustralia; ^2^Black Dog InstituteSydneyAustralia

**Keywords:** Internet therapy, e-therapy, cCBT, mental health, gay men, lesbian, minority stress, depression, anxiety, review

## Abstract

**Background:**

Lesbians and gay men have disproportionately high rates of depression and anxiety, and report lower satisfaction with treatments. In part, this may be because many health care options marginalize them by assuming heterosexuality, or misunderstand and fail to respond to the challenges specifically faced by these groups. E-therapies have particular potential to respond to the mental health needs of lesbians and gay men, but there is little research to determine whether they do so, or how they might be improved.

**Objective:**

We sought to examine the applicability of existing mental health e-therapies for lesbians and gay men.

**Methods:**

We reviewed 24 Web- and mobile phone-based e-therapies and assessed their performance in eight key areas, including the use of inclusive language and content and whether they addressed mental health stressors for lesbians and gay men, such as experiences of stigma related to their sexual orientation, coming out, and relationship issues that are specific to lesbians and gay men.

**Results:**

We found that e-therapies seldom addressed these stressors. Furthermore, 58% (14/24) of therapies contained instances that assumed or suggested the user was heterosexual, with instances especially prevalent among better-evidenced programs.

**Conclusions:**

Our findings, and a detailed review protocol presented in this article, may be used as guides for the future development of mental health e-therapies to better accommodate the needs of lesbians and gay men.

## Introduction

Over the past two decades, Internet self-help therapy, hereafter referred to as “e-therapy”, has rapidly become established as a recognized therapeutic approach in the treatment of depression and anxiety disorders [1-5]. E-therapy is also increasingly being delivered via mobile phone applications [6,7]. E-therapy’s effectiveness as a general mode of therapy is now supported by a considerable body of research [3,8-11]. Individual e-therapies within this health care mode have been tested to varying degrees. For example, MoodGym (Australia) has been examined in 17 research trials and seven randomized controlled trials [12], while some other currently available therapies have yet to be supported by research evidence.

E-therapies are an attractive option because they are relatively cost-effective [4,10,13-15], accessible, and able to maintain user anonymity [4,10,15]. In addition, e-therapies have been identified as particularly suitable for use by marginalized populations [13,16], such as rural persons [5] or same-sex attracted persons [5,16]. The use of e-therapies is now promoted by peak health care bodies, such as the National Health Service (NHS) in the United Kingdom [17,18], the Australian Psychological Society (APS) in Australia [3], and the Ministry of Health in New Zealand [19]. The NHS in particular has supported e-therapies by incorporating them into the United Kingdom’s health care strategy and subsidizing their use [17,20]. The net outcome is that e-therapies are not only on the rise, but are now recognized as an integral constituent of future health care solutions of Australia, the United Kingdom, and New Zealand, with therapies also common in many other regions, including the United States and parts of Europe [19,21].

While e-therapies are demonstrably an important tool in addressing mental health issues, most are designed for the population in general. Little work has been done to evaluate whether their content and language accounts for and meets the needs of individuals who identify as lesbian or gay. In Australia, homosexual and bisexual persons are three times as likely to experience depression and twice as likely to experience anxiety as the general population [22]. They are less likely to seek treatment, and when they do, they tend to have considerable concerns about experiencing discrimination [23]. Previous research, such as that grounded in Minority Stress Theory, has shown that health care systems often assume heterosexuality, and that this can have adverse mental health effects for same-sex attracted persons [24-26]. These negative effects not only arise from overt discrimination, such as doctors treating patients differently, but may also arise from the incongruence experienced by interacting with social structures that do not take the minority group into account, where the group is invisible to its language and design [27-29]. Thus, an e-therapy that replicates this incongruence might inadvertently contribute to minority stress, perhaps resulting in some lesbians and gay men disconnecting with a treatment.

Furthermore, it is insufficient for health care solutions to simply acknowledge sexual diversity. Lesbians and gay men are known to experience a unique set of mental health challenges that require tailored resources [29-32], such as dealing with discrimination and other forms of stigma, “coming out” to family and friends, and managing the process of concealing and disclosing their sexual orientation at work, in social settings, and other facets of life [28-31]. While avoiding language and content that assume heterosexuality on the part of the user is important, content that specifically targets these and other challenges is needed to sufficiently cater to the mental health needs of lesbians and gay men [29,33-36].

At present, it is not known the extent to which existing e-therapies avoid an assumption of heterosexuality in their language and other content (such as imagery depicting solely heterosexual persons and survey questions that could only be applicable to heterosexual partnering), or the degree to which they specifically address the mental health challenges of lesbians and gay men. This article responds to some of these questions by presenting findings from a review we conducted of a large number of widely accessible English language e-therapies. The main aim of the review was to provide an analysis of the current field of e-therapies with regard to accommodating the needs of lesbians and gay men and, in doing so, to identify areas that might be considered for improvement in the future development of programs. While some findings presented in this paper may be useful to tailoring content to the needs of other sexual minorities, such as bisexual, transgender, or asexual persons, it was decided that each of these groups is sufficiently different to hold unique sets of needs with regard to health and experiences of discrimination [37,38], and each merit a study tailored to them. Given that such a scope was beyond this project, we have focused our enquiry explicitly on the needs of lesbians and gay men as a starting point.

## Methods

### Selection of E-Therapies

We included both Web-based interventions and app-based interventions. A Web-based intervention is defined as “a primarily self-guided intervention program that is executed by means of a prescriptive online program operated through a website and used by consumers seeking health and mental health related assistance” [39]. An app-based intervention is essentially the same as a Web-based intervention except that it is operated through a mobile phone application using phone memory and/or the Internet.

Given that the number of Web-based interventions and app-based interventions is large and rapidly growing, we focused on those that were provided in English and were most likely to be effective and widely accessible for the prevention or treatment of depression and anxiety. To build this sample, we used four selection criteria; an intervention was included if it met all four criteria. First, the intervention needed to provide a recognized therapeutic modality, as categorized by the Centre for Mental Health Research, which refers to therapies that engender mood/behavior change via the application of evidence-based methods, such as cognitive behavior therapy (CBT), acceptance and commitment therapy (ACT), and Narrative Therapy. This eliminated general information websites, diary apps, and other such content that would merit a separate enquiry and that did not match our aim of scrutinizing structured therapies. Second, the intervention had to target depression and/or anxiety disorders. This encompassed generalized depression, bipolar disorder, post-traumatic stress disorder, generalized anxiety disorder, social anxiety disorder, panic disorder, obsessive-compulsive disorder, and social phobia. Third, the intervention had to be open access and therefore more easily accessible than paid therapies. Therapies that are free to the residents of a particular country, such as those subsidized by the NHS for citizens of the United Kingdom, were considered open access. Finally, the intervention needed to be in English. We did not have sufficient resources to translate non-English language e-therapies.

We used Beacon to source our sample. Beacon is a comprehensive database of e-health publications from around the world that is managed and regularly updated by the Centre for Mental Health Research at the Australian National University [12,40]. The database is compiled by a panel of experts in the field of mental e-health therapies and provides categorization and ranking of e-therapies based on expert evaluation from the panel. It stipulates classifications for all four of our selection criteria listed above: the disorder/s covered by each therapy, whether the therapy uses a recognized therapeutic modality, whether it is open access, and the language/s in which the therapy is delivered.

All Beacon-listed e-therapies were evaluated for selection on the basis of our selection criteria. The final sample was determined on 1^st^ November, 2013. In all, 28 e-therapies met the selection criteria. However, we were unable to obtain researcher access to analyze four of the selected e-therapies. Thus, our final sample consisted of 24 e-therapies, which comprised 20 Web-based interventions and 4 app-based intervention. Following final selection, a second check was performed on the Beacon database to ensure that no errors were made in the selection process.

### Procedure

Our measures can be divided into “attributes” and “content domains”. Attributes are classifying characteristics of e-therapies, such as their length, structure type, and whether they have research evidence to support their effectiveness. Content domains are measures to assess how appropriate therapies are for lesbians and gay men.

The Beacon database provided much of the attribute information for the 24 therapies in our sample. We focused on the length of the therapy (short, medium, long; as categorized by Beacon), extent of research evidence that supported the therapy’s effectiveness as measured by number of randomized controlled trials (RCTs) and other studies, country of origin of the therapy, and whether the therapy was a Web-based intervention or an app-based intervention. In addition, we also noted whether or not the e-therapy delivered content using scenarios: stories and examples involving characters. This last attribute was chosen given that e-therapies that contain scenarios have the added challenge of developing scenarios that are inclusive; for example, providing stories that are not limited to heterosexual relationships.

We then analyzed each therapy according to eight content domains (see Table 1). These domains cover key areas in which e-therapies might feasibly be tailored to accommodate or appeal to lesbians and gay men. They were derived from previous research that has identified inequalities and challenges faced by lesbians and gay men [16,27-29,35,36,41,42], including issues of lesbians and gay men feeling invisible or left out in health care provisions (eg, as a result of the practitioner overtly assuming they are heterosexual), and inadequate provision of resources that are suited to the needs and experiences of lesbians and gay men, which include coming out (eg, disclosing sexual identity to family or colleagues), same-sex relationships, and lack of appropriate references to helplines. Furthermore, we responded to a range of stigma-related challenges as described by Minority Stress Theory, such as discrimination, prejudice, fear of discrimination, and internalized stigma [25]. In this case, internalized stigma refers to lesbians or gay men internalizing or adopting negative attitudes that others may have about lesbians or gay men. We refer to these stigma-related challenges under the broad term “homonegativity”. We also analyzed whether therapies avoided instances that assumed or suggested the user was heterosexual. This involved assessing the appropriateness of language, such as whether a therapy referred to one’s “spouse”, which signifies marriage between a man and a woman. It also involved assessing the range of examples and scenarios used to deliver content within a therapy, and in particular whether all examples that indicated sexuality only depicted heterosexuality (eg, wives and husbands).

E-therapies were rated by the first author of this article on each content domain with a “yes” or “no”, according to the presence or absence of content. Detailed notes were also taken to justify the rating and to assist with checking inter-rater reliability. Results were recorded in a spreadsheet and paired with analytical notes and references to screenshots and excerpts, and the date when the analysis for each e-therapy was conducted. The reliability of ratings was checked by a second independent researcher, who was not otherwise involved in the research and is not an author of this article. This independent researcher rated 20% of the sample, totaling 5 Web-based interventions. A 97.5% agreement was reached between the original ratings and those of the independent researcher. There was only one disagreement, which was resolved easily through discussion and followed up by further relevant checking to ensure the error was an isolated one. In all, inter-rater reliability scores indicated a high level of confidence in the accuracy of the original ratings.

**Table 1:** Eight content domains used to assess the applicability of 24 e-therapies for lesbians and gay men.

	Content domains
1	Referred to lesbians and gay men in the introductory section of the therapy
2	Explicitly addressed homonegativity
3	Explicitly addressed coming out
4	Explicitly referred to same-sex relationships
5	Used imagery that depicted lesbians and/or gay men
6	Avoided instances that assumed or suggested the user was heterosexual
7	Provided references to mental health resources aimed at lesbians and gay men
8	Explicitly referred to lesbians and gay men in other ways not captured by the above

## Results

### Profile of E-Therapies

Table 2 summarizes attributes of the 24 e-therapies. A majority (67%, 16/24) were targeted to the treatment of depression and/or generalized anxiety disorder. Three-quarters (75%, 18/24) delivered content using scenarios. Just over half (54%, 13/24) were developed in Australia. This was in large part because fewer of those from other countries, particularly the United Kingdom and United States, were available for free and therefore these did not meet our criterion of open access. Half (50%, 12/24) were supported by research evidence and three of these were verified by randomized controlled trials. Almost all (92%, 22/24) therapies were categorized by Beacon as long.

**Table 2:** Profile of e-therapies (N=24).

E-therapy profile		n (%)
**Therapy type**		
	Web-based intervention	20 (83)
	App-based intervention	4 (17)
**Disorder type** ^a^		
	Depression	12 (50)
	Bipolar	0 (0)
	Generalized Anxiety Disorder	11 (46)
	Social Anxiety Disorder	3 (13)
	Panic Disorder	3 (13)
	Post-Traumatic Stress Disorder	4 (17)
	Phobia	2 (8)
	Obsessive Compulsive Disorder	1 (4)
**Content delivery method**		
	Scenario	18 (75)
	Non-scenario	6 (25)
**Origin**		
	Australia	13 (54)
	United States	5 (21)
	United Kingdom	3 (13)
	New Zealand	1 (4)
	United States/Canada^b^	1 (4)
	Israel	1 (4)
**Evidence rating (x/5)** ^c^		
	Two or more	3 (13)
	One	9 (38)
	Zero	12 (50)
**Length** ^d^		
	Long	22 (92)
	Moderate	2 (8)
	Short	0 (0)

^a^Therapies may cater to multiple disorders, therefore total exceeds N=24 (100%).

^b^Joint collaboration between United States and Canada.

^c^Evidence rating is the score awarded by Beacon to indicate the degree to which an e-therapy is supported by research evidence. Zero indicates no evidence or no evidence of effectiveness. One indicates some evidence but no evidence from randomized controlled trials. Two or higher indicates evidence of effectiveness, including from randomized controlled trials.

^d^Long: 5+ modules; Moderate: 3-5 modules; Short: 1-2 modules.

### Overall Ratings

Table 3 displays numbers and percentages of therapies that scored positively in the eight content domains that we examined in our review. In all, few therapies scored in content domains 1-5. These domains concerned topics of coming out, homonegativity, same-sex relationships, visually depicting lesbians and/or gay men, and acknowledging lesbians and gay men in the introductory sections of a therapy. Examples of this content included forum articles discussing experiences of coming out to parents, dealing with sexuality-based abuse, and experiences of entering the same-sex dating scene. Four (17%, 4/24) therapies were found to have references to mental health resources aimed at lesbians and gay men. In all cases, these comprised a list of crisis referral services aimed at lesbians, gay men, and other sexual minorities. Ten therapies (42%, 10/24) were found to avoid instances that assumed or suggested users were heterosexual. Instances where e-therapies that failed to do this included referring to a partner as a “spouse” (a term that typically denotes heterosexual partnering) and imagery that depicted only heterosexual relationships and/or nuclear families.

One e-therapy, Big White Wall (BWW), accounted for the majority of domain scores. BWW scored in domains 1-5 and 8, while none of the other therapies scored in more than two content domains. BWW is comprised of both professionally-created and user-created content. The latter was largely responsible for the positive scores. Professional content actually failed to avoid instances that assumed user heterosexuality and only scored positively in one other content domain, when it mentioned same-sex attracted persons twice: in an interview transcript about “managing your differences” and in a review of a book that included a gay character. The count of user-generated same-sex orientation specific content was comparably enormous. When we entered keywords into BWW’s search box on the 20th November, we found 120 artworks and 129 forum results for the word “gay” and 56 and 57 respectively for the word “lesbian”. Other keywords such as “queer” and “homosexual” returned a smaller number of results. The primarily inclusive nature of these posts were confirmed on closer scrutiny, which revealed content about coming out, family issues relating to sexuality, inclusivity, resilience, artworks with rainbow flags, gay couples, and even a picture protesting against Uganda’s recent anti-homosexuality Act.

**Table 3:** Numbers and percentages of e-therapies that scored in each content domain (N=24).

	Content domains	Yes, n (%)
1	Referred to lesbians and gay men in the introductory section of the therapy	0 (0)
2	Explicitly addressed homonegativity	1 (4)
3	Explicitly addressed coming out	2 (8)
4	Explicitly referred to same-sex relationships	1 (4)
5	Used imagery that depicted lesbians and/or gay men	2 (8)
6	Avoided instances that assumed or suggested the user was heterosexual	10 (42)
7	Provided references to mental health resources aimed at lesbians and gay men	4 (17)
8	Explicitly referred to lesbians and gay men in other ways not captured by the above	1 (4)

### Ratings According to Content Delivery Method and Evidence Rating

Given that e-therapies that use scenarios (eg, stories involving characters) have the added challenge of developing scenarios that are inclusive of marginalized populations, and may be more appealing to users, we examined whether e-therapies that scored in each of the eight domains were among those that used scenarios. All of the therapies that had content in domains 1-5 and 7-8 were found to use scenarios. Thus, none of the non-scenario therapies scored in these domains. In contrast, only four of the 10 therapies that scored in content domain 6 (ie, avoided instances that assumed user heterosexuality) used scenarios while the remaining six therapies did not use scenarios. Thus, all non-scenario therapies scored in this domain.

Assuming that better-evidenced e-therapies are also more likely to appeal to users, we further examined whether therapies that scored in each of the eight content domains were among those with research evidence. In all, therapies that scored in the content domains were mostly those supported by research evidence. In particular, all of the therapies that had content in domains 1-5 and 7-8 had an evidence rating of 1 or 2, which means that they were supported by at least some research evidence. Of the 10 therapies that scored in content domain 6 (ie, avoided instances that assumed user heterosexuality), five (50%) had an evidence rating of 1 or 2 and the remaining five (50%) had an evidence rating of zero. Thus, e-therapies with an evidence rating of zero did not score in any of the content domains except for content domain 6. The three e-therapies with an evidence rating of 2 or higher (ie, were supported by evidence from randomized controlled trials) each scored in one domain, with one scoring in domain 1, one in domain 5, and one in domain 6.

As suggested by the above results, there was considerable overlap between therapies that were supported by research evidence and those that used scenarios. Specifically, of the 18 e-therapies that used scenarios, three (17%) had evidence ratings of 2 or higher, eight a rating of 1 (44%), and seven (39%) a rating of zero. Of the six e-therapies that did not use scenarios, none had evidence ratings of 2 or higher, only one (17%) had an evidence rating of 1, and five (83%) had a rating of zero.

## Discussion

### Principal Findings

Overall, e-therapies seldom catered to the needs of lesbians and gay men. Most did not include content that explicitly covered key experiences such as coming out or being in a same-sex relationship. For the most part, the language used did not account for same-sex attracted clients. Further, more than half the e-therapies used content that assumed or suggested user heterosexuality. It would appear that the experiences of lesbians and gay men were seldom considered in the development of these e-therapies. This corroborates with past research in this area, which has found that no existing computerized cognitive behavioral therapy (cCBT) programs address challenges specific to lesbians and gay men [16]. It also reinforces broader work that demonstrates a shortage of mental health care that caters to the needs of same-sex attracted populations [16,28,31]. This is despite a comprehensive and growing body of research that shows that therapy modes that fail to do so can alienate lesbians and gay men and lead to diminished therapeutic outcomes [27,29-31,34].

Interestingly, whether or not a therapy used scenarios (ie, stories and examples involving other characters) resulted in markedly different content domain scores. All of the non-scenario therapies did not assume sexual orientation in their language and content, but they also did not record a positive score across any of the other content domains. On the other hand, only 42% of scenario therapies avoided instances that assumed user heterosexuality, but some scored positively in other content domains. This is attributable to distinctions in the structural characteristics of the two therapy types. All therapies—both scenario and non-scenario—were careful to address their audience in neutral language, to cater to both sexes if nothing else. The locus of content that assumed heterosexuality was in the extra layer of content in scenario therapies. These scenarios included stories about nuclear families, heterosexual dating, and so forth. Audio-visual content often exemplified the text and was also problematic. This content tended to be prominent and pervasive in therapies and failed to present non-heterosexual alternatives. Non-scenario therapies avoided the issue of assuming heterosexuality by not having this layer. They also tended to present content in a broader way; where a scenario therapy may discuss the financial strain on John’s marriage, a non-scenario therapy may discuss “building positive relationships”. Issues such as coming out or homonegativity were too narrow to fall within the scope of the latter, just as content specifically about heterosexual relationships was too narrow to be explicitly addressed. This contrasted with scenario therapies, which frequently covered heterosexual issues and addressed specific problems such as dating or partnering. Thus, on the one hand, non-scenario therapies expressed content broadly, which had the effect of not excluding specific sexualities but also not dealing with sexuality-specific content. On the other hand, scenario therapies dealt with sexuality-specific content but largely omitted same-sex attraction.

In our sample, the scenario therapies were more common and scored better on Beacon ratings of research evidence than non-scenario therapies. In fact, the majority were supported by research evidence, as compared to only one-sixth of non-scenario therapies. Scenario therapies appear to be the more prominent therapy type, certainly within our sample, and given that individuals are more likely to be referred to evidence-based e-therapies by health professionals, the low content domain scores and high frequency of heteronormative language in scenario therapies may need attention in future development of programs.

As our results show, only one to two therapies included content that specifically addressed mental health challenges experienced by lesbians and gay men. Big White Wall (BWW) is the program that accounted for the majority of these cases. BWW is a UK-based therapy designed to help treat stress, depression, and general anxiety. Beacon described it as a service that “utilizes the principles of social networking” [12]. This is because while BWW offers a range of professionally created self-help material and guided activities, it also integrates user-created social support groups composed of BWW clients. They can start forum discussions and express themselves through art and writing. The convergence of professional and user-generated content is integral in BWW, which is composed of polygenic content organized in a non-linear structure. This differentiates it from major therapies like MoodGym, Beating the Blues, and AnxietyOnline. Such a format may be significant for lesbians and gay men, as positive networks have been identified as core resilience drivers for same-sex attracted persons [43-45].

BWW’s positive scores, and the fact that user content was largely responsible, highlights two important points. First, the level of user posts pertaining to same-sex attraction demonstrates the merit of incorporating social network-styled structures into e-therapies to assist in catering to lesbians and gay men. For the many therapies that cater to the general population, this may be an efficient way to provide at least some content that targets lesbians and gay men. However, the second point is that the stark contrast between the level of professional and user-generated content illustrates a disparity between supply and demand for content that is specific to lesbians and gay men. The volume of topics raised, many of which paralleled our content domains, demonstrate a need by lesbians and gay men for information and guidance that is not currently being delivered by professional content. It also highlights that such issues as coming out, family acceptance, and inclusivity are important parts of the mental health experiences of lesbians and gay men, and there is a demand for therapies to address them. One of the particular advantages of e-therapies as a delivery mode is their capacity to deliver tailored, targeted content, perhaps using adaptive logic. Harnessing this capacity by targeting specific content to a user according to their sexual orientation is one way of catering to lesbians and gay men within e-therapies, but so far this appears to have been underutilized.

### Limitations and Future Directions

Findings of this review are limited to e-therapies we selected. While we chose selection criteria that enabled a diverse sample of e-therapies, they were nonetheless limited to those that used therapeutic modalities (eg, CBT, ACT), targeted depression and anxiety, were open access, and were in English. Language in particular is a notable limitation, as there are many instances of e-therapies in other languages. That said, we are confident that our sample was large and diverse enough for our findings to be broadly applicable to e-therapies, at least in English-speaking countries.

Our findings were also limited to e-therapies that were available on November 2013. The e-therapy landscape is changing rapidly, with existing e-therapies constantly being modified and new therapies being developed. For example, some e-therapies that would have fit our selection criteria were not included in the analysis because they were in research trial phases at the time. It should be noted that SPARX—an adventure computer game that treats depression—has recently been trialed in a “Rainbow” version for adolescents who are same-sex attracted or questioning [16,46]. This therapy was not available at the time of our analysis, and is only available on CD-ROM and not currently available online. App-based interventions are propagating even more quickly, and we suspect that at the time of publication of this article there will be a significantly larger number of app-based interventions that might have fit our selection criteria. Finally, our findings and review protocol are limited to lesbians and gay men. We felt this limitation was necessary because issues faced by other sexual minorities, such as people who identify as bisexual or pansexual, are often different compared to lesbians and gay men [23,37,38] and may therefore need to be handled somewhat differently in e-therapies. In future, studies are needed to examine ways in which e-therapies might be further improved to accommodate the specific needs of other sexual minorities.

Despite its limitations, our work drew a relevant sketch of the deficiencies of current e-therapies in catering to lesbians and gay men, and may help to guide future projects to improve this. On the whole, it is clear that greater attention to the needs of lesbians and gay men is desirable in the design of e-therapies. Consideration should be given to the construction of language—verbal and audio-visual—that does not preclude the experiences of same-sex attracted persons. We acknowledge that developers of e-therapies that are intended for a general audience may be concerned that adding content specifically tailored to lesbians and gay men might be off-putting to predominantly heterosexual users. Tailoring to all eight of our content domains may not be appropriate for all therapies, but it may be valuable to consider whether minor changes such as including gay or lesbian-specific helplines or some acknowledgement of same-sex attracted persons may make the e-therapy more attractive for these populations. As noted earlier, using adaptive logic to tailor content to a user’s sexual identity could be a particularly powerful way of ensuring content meets the needs of specific sexual identity groups without deterring other groups. In fact, our review protocol could be useful to developers as a tool to assess the inclusivity of their therapeutic programs, by repeating the steps outlined in our methodology to conduct a review of their own e-therapy and to identify potential areas where content could be improved or tailored. In terms of future research, we believe that further inquiry is needed from the perspectives of same-sex attracted persons to identify specific kinds of content that would make them feel more included and that address the specific mental health needs of these populations.

### Conclusions

In this article, we presented the findings of a review of Web-based and app-based interventions for the prevention and treatment of depression and anxiety with regard to the degree to which they catered to lesbians and gay men. The majority did not address many of the key additional factors for depression and anxiety experienced by lesbians and gay men. They largely did not acknowledge lesbians and gay men, or address core issues like coming out, dealing with discrimination and prejudice, or same-sex relationships. Many of the therapies that used scenarios to deliver content, which tended to be the more prominent type of e-therapy in terms of numbers and evidence of effectiveness, also contained instances of language and content that assumed user heterosexuality. As we have outlined, past research indicates that many therapies may exclude lesbians and gay men and in doing so may inadvertently contribute to minority stress. This is particularly an issue given that these populations already experience comparatively high rates of mental health problems, are less likely to engage with therapy, and are known to express concerns around fears of discrimination. Our findings suggest that e-therapies could do more to address the needs of lesbians and gay men, thus enabling these populations to benefit more greatly from the rise of e-therapy for depression and anxiety and to therefore contribute to reducing disparities in mental health between heterosexual and non-heterosexual populations. To this end, we suggest that the review protocol developed for this study could be utilized by developers to help monitor and improve the applicability of e-therapies for lesbians and gay men.

## Abbreviations

ACT: acceptance and commitment therapy
APS: Australian Psychological Society
BWW: Big White Wall e-therapy
CBT: cognitive behavior therapy
CCBT: computerized cognitive behavioral therapy
NHS: National Health Service
RCT: randomized controlled trial
